# Chemical vapor deposition growth of carbon nanotube confined nickel sulfides from porous electrospun carbon nanofibers and their superior lithium storage properties†

**DOI:** 10.1039/c8na00234g

**Published:** 2018-10-12

**Authors:** An Wang, Sanmu Xie, Rong Zhang, Yiyi She, Chuan Chen, Micheal K. H. Leung, Chunming Niu, Hongkang Wang

**Affiliations:** State Key Lab of Electrical Insulation and Power Equipment, Center of Nanomaterials for Renewable Energy (CNRE), School of Electrical Engineering, Xi'an Jiaotong University Xi'an 710049 People's Republic of China hongkang.wang@mail.xjtu.edu.cn; Ability R&D Energy Research Centre (AERC), School of Energy and Environment, City University of Hong Kong Hong Kong SAR People's Republic of China; Global Energy Interconnection Research Institute Co., Ltd. Future Science Park, Changping District Beijing 102211 People's Republic of China

## Abstract

Multidimensional architecture design is a promising strategy to explore unique physicochemical characteristics by synergistically integrating different structural and compositional materials. Herein, we report the facile synthesis of a novel dendritic hybrid architecture, where carbon nanotubes (CNTs) with nickel sulfide nanoparticles encapsulated inside are epitaxially grown out of the porous electrospun N-doped carbon nanofibers (CNFs) (denoted as CNT@NS@CNFs) through a combined strategy of electrospinning and chemical vapor deposition (CVD). The adopted thiophene (C_4_H_4_S) not only serves as a carbon source for the growth of CNTs but also as a sulfur source for the sulfurization of Ni particles and S-doping into carbon matrices. When examined as an anode material for lithium-ion batteries (LIBs), the dendritic CNT@NS@CNFs display superior lithium storage properties including good cycle stability and high rate capability, delivering a high reversible capacity of 630 mA h g^−1^ at 100 mA g^−1^ after 200 cycles and 277 mA h g^−1^ at a high rate of 1000 mA g^−1^. These outstanding electrochemical properties can be attributed to the novel hybrid architecture, in which the encapsulation of nickel sulfide nanoparticles within the CNT/CNFs not only efficiently buffers the volume changes upon lithiation/delithiation, but also facilitates charge transfer and electrolyte diffusion owing to the highly conductive networks with open frame structures.

## Abstract 2

Carbon-nanotube confined nickel sulfides grown on porous carbon nanofibers were prepared *via* an electrospinning-CVD method and showed superior lithium storage performance.

## Graphical abstract

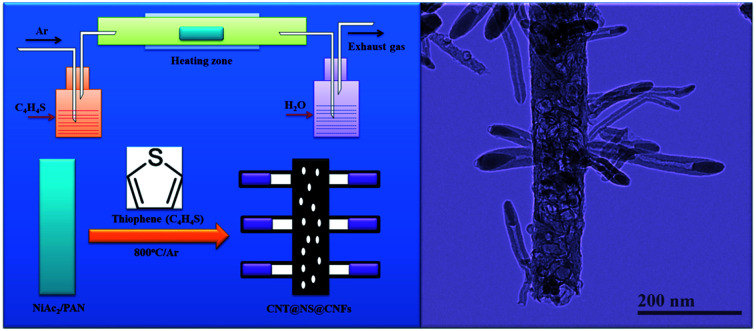

## 1. Introduction

As a promising rechargeable energy storage device, lithium-ion batteries (LIBs) have been widely used in many fields including portable electronic devices and electric vehicles due to their high-energy density, long cycle life and environmental friendliness as compared to other commercial batteries.^1–6^ However, the graphite anodes commonly used in current commercial LIBs remain insufficient for emerging applications in renewable energy storage systems, owing to the low theoretical specific capacity of graphite (372 mA h g^−1^).^7,8^ Moreover, the low lithium insertion/extraction voltage (below 0.2 V *vs.* Li^+^/Li) also causes safety problems owing to the formation of lithium dendrites.^5^

In order to overcome these drawbacks, substantial research efforts have been made to explore new anode materials with higher specific capacity. Novel carbon nanostructures such as carbon nanotubes (CNTs), graphene and carbon nanofibers (CNFs) have attracted considerable attention, owing to their large specific areas and the resultant large lithium storage sites.^9–11^ In particular, the carbon-based materials with intercalation–deintercalation mechanisms exhibit minor volume changes and show excellent electrochemical performance owing to their high electronic conductivity and superior mechanical properties.^9,12–15^ In addition, doping carbonaceous materials with exotic elements such as sulfur and nitrogen can also enhance the lithium storage performance, but still with limitations.^16–19^

Recently, nickel sulfides (*e.g.*, NiS, NiS_2_, Ni_3_S_2_, and Ni_9_S_8_) have been proposed as alternative anodes in LIBs owing to their high capacities achieved *via* a conversion mechanism.^15,17,20–22^ Among them, Ni_3_S_2_, one of the most important phases of nickel sulfide, is abundant in nature and cheap and has been reported to exhibit unique properties for electrochemical energy storage and conversion in recent years.^18,23–25^ However, when used as the anode in LIBs, Ni_3_S_2_ suffers from poor electrical conductivity and large volume changes. Thus, hybridizing nickel sulfides with carbon-based nanostructures has been suggested as a potential strategy to overcome these limitations.^13,23^

Herein, we demonstrate the facile synthesis of a unique hierarchical architecture containing carbon nanotubes (CNTs), carbon nanofibers (CNFs) and nickel sulfide (NS) nanoparticles, in which CNT confined NS particles are directly grown from porous N-doped CNFs (denoted as CNT@NS@CNFs) *via* a combined electrospinning and chemical vapor deposition method. When used as anode materials for LIBs, the dendritic CNT@NS@CNFs demonstrated superior cycling stability and rate capability, owing to the advantageous structural characteristics.

## 2. Experimental section

### Materials preparation

All the chemicals including polyacrylonitrile (PAN, MW = 150 000, Macklin), nickel acetate (NiAc_2_·4H_2_O, Alfa Aesar), and dimethylformamide (DMF, Macklin) were used as received without any further treatment. In a typical synthesis of CNT@NS@CNFs, 0.8 g polyacrylonitrile was dissolved in 10 mL DMF under vigorous stirring until a clear transparent solution was obtained. Then 1.0 g Ni(Ac)_2_·4H_2_O was added and the solution was kept under stirring for 2 h, and then a light-green precursor solution was obtained. For electrospinning, the flow rate was set at 0.5 mL h^−1^ and aluminum foil was used to collect the fibers. The distance between the needle and the fiber collector was set at around 15 cm and a high voltage of 20 kV was applied to initiate the electrospinning. The obtained fibers were first stabilized under an Ar atmosphere at 150 °C for 1 h and then heated to 800 °C at a ramp rate of 10 °C min^−1^. When the temperature remained constant at 800 °C, thiophene was introduced into the horizontal tubular quartz reactor by bubbling under Ar gas with a flow rate of 100 mL min^−1^ at room temperature. After maintaining the temperature at 800 °C for 30 min, thiophene was removed, and the tube was naturally cooled down to room temperature under an Ar flow; thus the final products were obtained.

### Structural characterization

The phase structure of the product was identified by powder X-ray diffraction (XRD) using a Bruker D2 PHASER diffractometer. Morphologies were characterized by scanning electron microscopy (SEM, FEI Quanta 250F) and transmission electron microscopy (JEOL JEM-2100). The Raman spectrum was obtained using a microscope (Renishaw RE01) with a laser wavelength of 633 nm. Thermogravimetric analysis (TGA) was carried out using a Mettler Toledo thermal analyzer at a heating rate of 10 °C min^−1^ in the range of 25–800 °C in air. The surface area and pore features were characterized on a Quantachrome Autosorb IQ analyzer. The surface composition and chemical states of the product were determined by X-ray photoelectron spectroscopy (XPS, Thermo Fisher ESCALAB Xi^+^). All the binding energies were calibrated using the reference C 1s peak at 284.6 eV.

### Electrochemical measurements

The electrochemical properties were examined by using CR2025 coin-type cells assembled in an Ar-filled glovebox (both H_2_O and O_2_ content less than 1.0 ppm). The working electrode was prepared by mixing the active materials, carbon black (Super-P) and polyvinylidene difluoride (PVDF) in a weight ratio of 8 : 1 : 1, which was then cast on Cu foil, followed by drying in a vacuum at 70 °C overnight. Lithium foil was used as both the counter electrode and reference electrode, a Celgard 2400 microporous membrane was used as the separator and 1 M LiPF_6_ in ethylene carbonate/dimethyl carbonate (1 : 1 in volume) was used as the electrolyte. The galvanostatic charge–discharge tests were performed on a battery test system (Neware BTS, China) in the range of 0.01–3.0 V at room temperature (25 °C). Cyclic voltammetry (CV) was performed on an Autolab PGSTAT 302N electrochemical station in the range of 0.01–3.0 V at a scan rate of 0.2 mV s^−1^. Electrochemical impedance spectroscopy (EIS) was examined by applying a voltage amplitude of 10 mV in the frequency range of 10 MHz to 0.01 Hz. The specific capacities were calculated based on the weight of the CNT@NS@CNFs nanocomposites.

## 3. Results and discussion

**Fig. 1:**
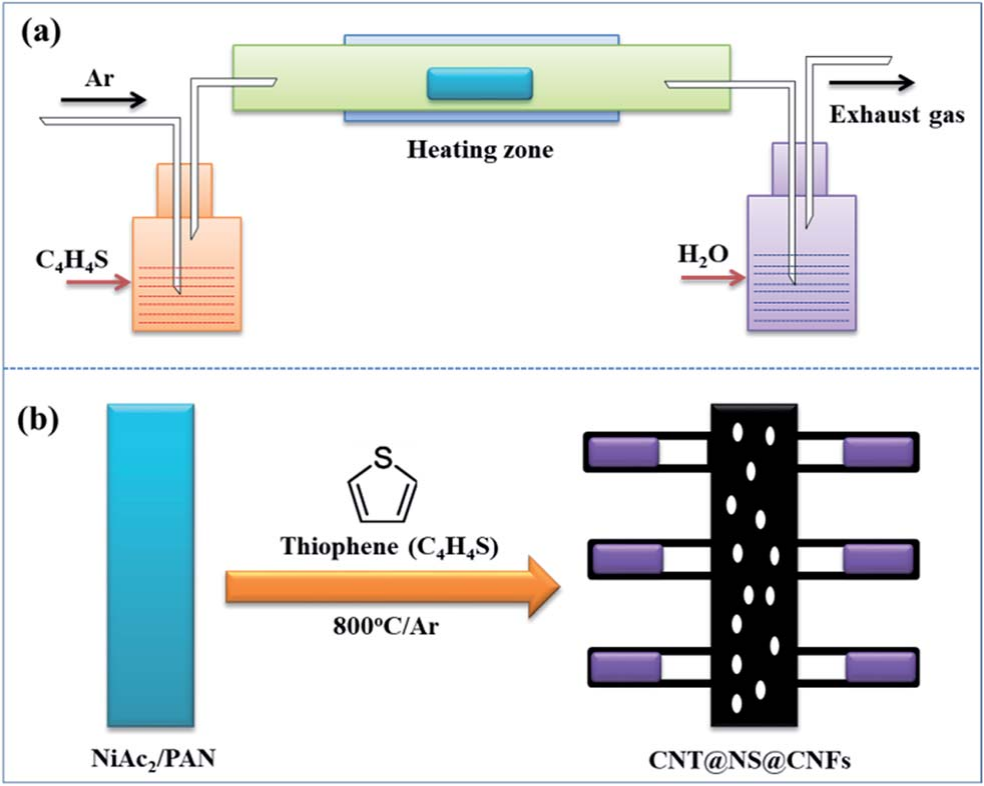
Schematic illustrations of (a) the synthetic approach and setup, and (b) the structural evolution from electrospun NiAc_2_/PAN precursor nanofibers to the dendritic CNT@NS@CNF hybrid architecture.

The dendritic CNT@NS@CNF hierarchical architecture is synthesized *via* a combined electrospinning and chemical vapor deposition (CVD) method. The precursor nanofibers containing polyacrylonitrile (PAN) and nickel acetate (NiAc_2_) (denoted as PAN/NiAc_2_) are prepared through electrospinning, which is an effective strategy for the fabrication of CNFs.^26^Fig. 1a shows the schematic illustration of the CVD setup and the reaction process, while Fig. 1b shows the structural evolution of PAN/NiAc_2_ during the annealing and CVD processes. During annealing in an Ar atmosphere, PAN with a high N content (∼26.4 wt%) carbonizes into N-doped carbon, which serves as a reducing agent to convert nickel salt into metallic Ni nanoparticles. It's worth mentioning that the transition metal particles can catalyze graphitic crystallization.^9,27,28^ When introducing thiophene (C_4_H_4_S) at 800 °C, the thiophene decomposes and produces a large amount of H_2_S and gaseous hydrocarbons ((CH_2_)_*x*_), which serve as sulfur and carbon sources, respectively. Under the catalytic effect of Ni nanoparticles (should be in a molten state) at high temperature, CNTs are epitaxially grown out of CNFs with the thiophene derived gaseous hydrocarbons as the carbon source. Simultaneously, the Ni particles react with H_2_S to form nickel sulfides according to the “dissolution–precipitation” mechanism.^18,29^ Thus the chemical reactions can be expressed as follows:

1 C_4_H_4_S → H_2_S + (CH_2_)_*x*_

2 H_2_S + Ni → Ni_3_S_2_ + H_2_

3
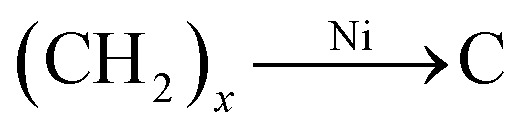


**Fig. 2:**
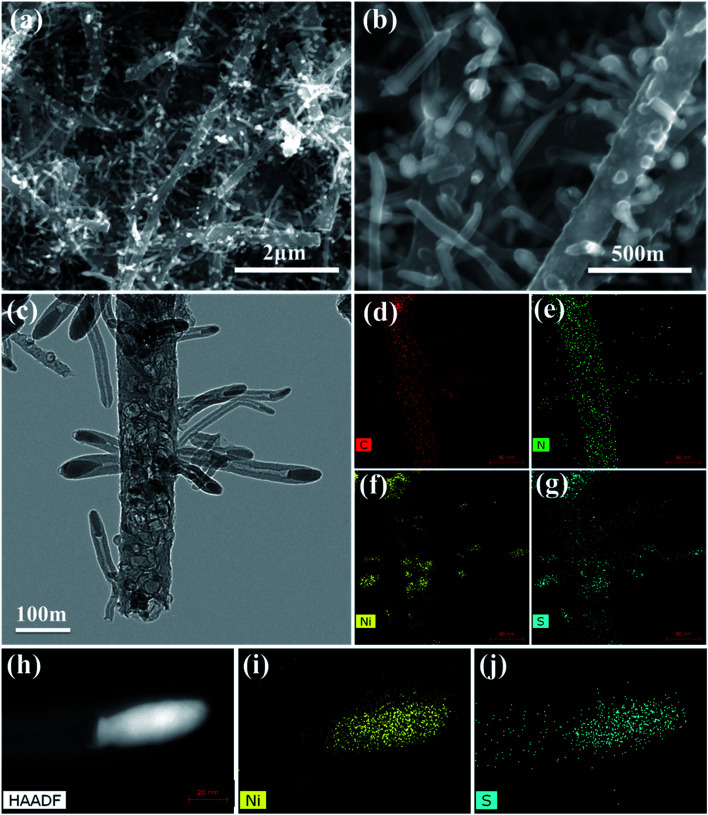
(a and b) SEM images, (c) TEM image and (d–g) the corresponding EDS elemental maps of (d) C, (e) N, (f) Ni and (g) S elements in the CNT@NS@CNF hybrid. (h) HAADF image of a branch of a CNT confined nickel sulfide nanoparticle with the corresponding EDS maps of (i) Ni and (j) S.

The morphological structures of the as-prepared CNT@NS@CNFs were investigated by SEM and TEM analyses. As shown in Fig. 2a and b, the electrospun 1D carbon nanofiber (CNF) backbones with lengths on the microscale and diameters of 200–300 nm are coupled with many branch-like CNTs, which are epitaxially grown out of the CNFs. In the TEM image (Fig. 2c), it's clearly observed that nickel sulfide nanoparticles are encapsulated inside the CNTs and locate at their tips. The CNTs are dozens to hundreds of nanometers in length and 20–50 nm in diameter. The elemental distribution was examined by energy-dispersive X-ray spectroscopy (EDS), and Fig. 2d–g show the overall elemental distribution within a single dendritic CNT@NS@CNF, where the C, N and S elements are uniformly distributed within the dendritic structure, which reveals the co-doping of N and S into the carbon frameworks. Fig. 2h shows the high-angle annular dark-field (HAADF) scanning transmission electron microscopy (STEM) image of a single CNT confined nickel sulfide nanoparticle, and the corresponding Ni and S elemental maps reveal the formation of nickel sulfide as well as the S-doping into CNTs, as the Ni element is only present at the tip while the S element mainly accumulates at the tip but is also present along the CNT. However, there is little N-doping into the CNTs, as they are produced by the CVD method using thiophene as the carbon source, while the N-doping into the CNFs arise from the carbonization of N-containing PAN.^9^

**Fig. 3:**
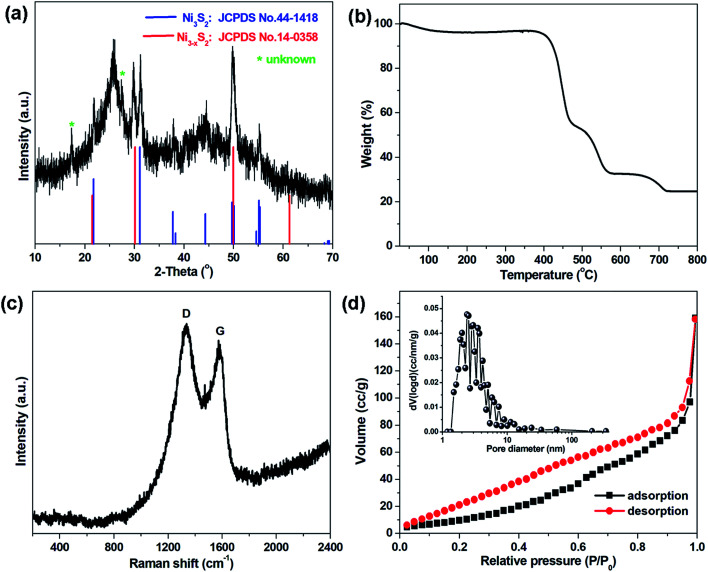
(a) XRD pattern, (b) TGA curve, (c) Raman spectrum, and (d) nitrogen adsorption–desorption isotherm with the inset showing the corresponding pore size distribution of CNT@NS@CNFs.

Fig. 3a shows the XRD pattern of the CNT@NS@CNF nanocomposites. The broad diffraction peak at around 26° can be assigned to graphitic CNTs and CNFs in the hybrid CNT@NS@CNFs. The other obvious peaks match well with crystalline Ni_3_S_2_ (JCPDS no. 44-1418) and crystalline Ni_3−*x*_S_2_ (JCPDS no. 140358). In addition, two peaks located at 17.3° and 27.6° are not well indexed, which may be related to some unknown NiS_*x*_ with different stoichiometry. Thermogravimetric analysis (TGA) was also performed to determine the relative content of carbon and nickel sulfides. As shown in the TGA curve of the hybrid (Fig. 3b), the total weight loss is around 73.5 wt%, accompanied by a 47.0 wt% weight loss in the range of 475–500 °C and then a 20.2 wt% weight loss in the range of 570–660 °C. The minor weight loss before 150 °C may be attributed to the evaporation of adsorbed water in the nanocomposite,^30^ while the weight loss at around 480 °C can be attributed to the combustion of the carbon, indicating that the carbon content is around 47.0%. On further increasing the temperature, the weight loss is due to the oxidation of nickel sulfides (*e.g.*, Ni_3_S_2_) into NiO (Fig. S1†).

Fig. 3c shows the Raman spectrum of the CNT@NS@CNF composites in the range of 200–2400 cm^−1^. Two obvious peaks at around 1340 cm^−1^ and 1580 cm^−1^ are assigned to the D-band and G-band, corresponding to the disordered carbon and the ordered graphitic carbon,^17,31^ respectively. The D-band reflects the defects in carbon-based materials associated with vacancies and amorphous carbon species, while the G peak originating from the stretching motion of the sp^2^ carbon pairs corresponds to the ordered graphitic carbon species.^13,18^ The intensity ratio of the D-band to the G-band (*I*_D_/*I*_G_) is 1.079, indicating a high defect degree of the hybrid material, which is beneficial for Li-ion storage in the graphene-based anodes.^13^ The increase in the disorder may be attributed to the influence of the embedded Ni_3_S_2_ nanoparticles and the defects introduced by N-doping and S-doping.^18,32^ Besides, no apparent peaks for Ni_3_S_2_ can be observed, which may be attributed to its full incorporation into the graphite matrix. The surface area, as well as the pore size features of the CNT@NS@CNFs, were characterized by N_2_ sorption isotherms (Fig. 3d), and the hybrid exhibits a specific surface area of 44.6 m^2^ g^−1^. The pore size distribution plot reveals the porous structure of the hybrid, consisting of mesopores with a total pore volume of 0.29 cm^3^ g^−1^, which is consistent with the SEM and TEM results.

**Fig. 4:**
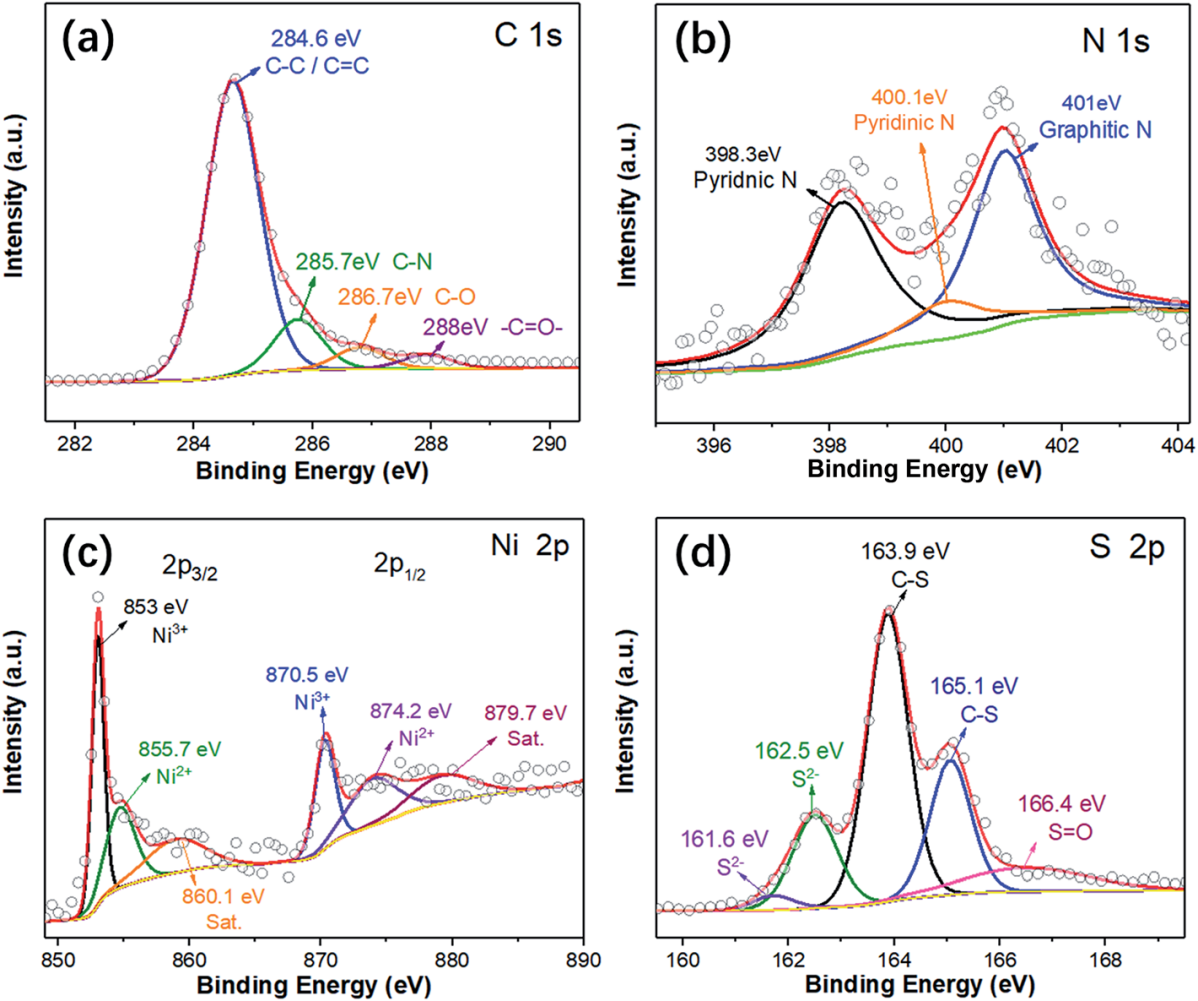
High-resolution XPS spectra of (a) C 1s, (b) N 1s, (c) Ni 2p, and (d) S 2p of the CNT@NS@CNFs.

In order to investigate the elemental compositions and the chemical states in the CNT@NS@CNFs, X-ray photoelectron spectroscopy (XPS) was performed, and the survey XPS spectrum clearly confirms the presence of Ni, S, C, N and O elements (Fig. S2†). As shown in Fig. 4a, the high-resolution C 1s XPS spectrum can be deconvoluted into four peaks, which can be correspondingly indexed to C–C (284.6 eV), C–N (285.7 eV), C–O (286.7 eV) and –C=O– (288 eV). In the N 1s XPS spectrum (Fig. 4b), three deconvoluted peaks located at 401, 400.1 and 398.3 eV can be assigned to the graphitic, pyrrolic and pyridinic types of N atoms.^13,17,33^ It's noteworthy that the existence of pyridinic N can increase electron conduction, thus facilitating the improvement of the rate performance of the electrodes. In addition, the N heteroatoms can help to enhance the whole architectural stability and induce more defects, disorder and a lower degree of crystallinity, which can create more Li^+^ storage sites and thus enhance capacity.^17,19^ In the Ni 2p XPS spectrum, the peaks at 855.7/874.2 eV and 853/870.5 eV correspond to Ni 2p_1/2_ and Ni 2p_3/2_, respectively, indicating the formation of Ni^2+^ and Ni^3+^.^10,24^ The satellite peaks at 860.1 and 879.7 eV are related to the higher energy states of electrons in the valence band, which is called a “shake-up” process.^34^ Furthermore, the peak located at 853 eV was considered as the characteristic peak of Ni_3_S_2_. In the S 2p region, the peaks at 161.6 and 162.5 eV are assigned to the S 2p_3/2_ and S 2p_1/2_ orbitals associated with Ni–S bonding.^35,36^ Besides, the main peaks at 163.9 and 165.1 eV can be attributed to the C–S bonds while the peak at 166.4 eV is ascribed to the oxidation of sulfur outside the surface.^13,35^

**Fig. 5:**
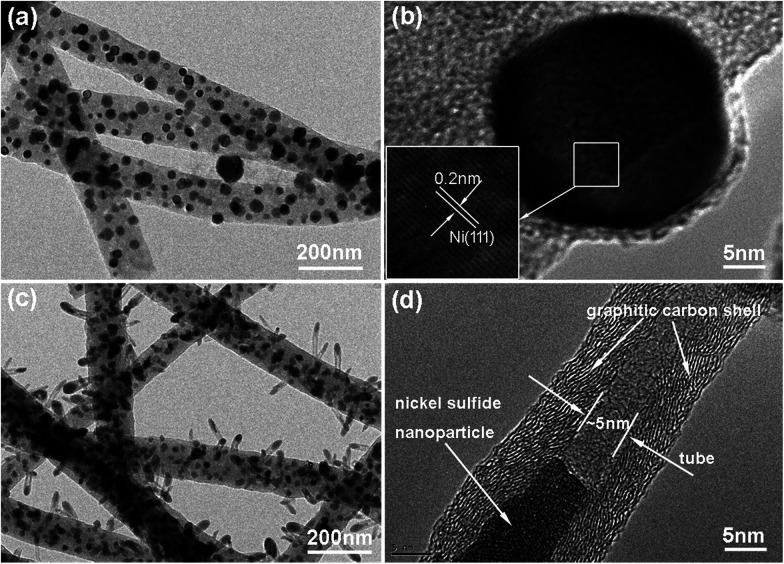
(a and c) TEM and (b and d) HRTEM images of the Ni@CNFs (a and b) obtained by annealing NiAc_2_/PAN at 800 °C without the presence of thiophene, and the intermediate CNT@NS@CNFs (c and d) obtained by introducing thiophene for 10 min at 800 °C.

Time-dependent experiments were also performed in order to understand the structural evolution during the CVD reaction. Fig. 5a shows the TEM image of the carbon nanofibers with encapsulated Ni nanoparticles (denoted as Ni@CNFs), which were obtained by annealing the NiAc_2_/PAN precursor nanofibers up to 800 °C and naturally cooling immediately. Apparently, there were no CNTs grown on the CNFs (Fig. S3†), indicating that the CNTs in the CNT@NS@CNFs are derived from the decomposition of thiophene under the catalytic effect of metallic Ni. In the HRTEM image (Fig. 5b), a Ni nanoparticle with a size of tens of nanometers shows clear lattice fringes with a *d* spacing of 0.2 nm, which can be indexed to the (111) plane of the Ni phase (JCPDS no. 04-0850). In contrast, when the temperature was ramped up to 800 °C and then thiophene was introduced for 10 min (Fig. 5c), CNT-capped nickel sulfides grew from the CNFs, and the nanoparticles are apparently observed and embedded within the CNTs, but are smaller than those in the Ni@CNFs. This can be explained by the fact that some of the Ni particles reacted with thiophene forming nickel sulfides. Fig. 5d shows the HRTEM image of a CNT with an encapsulated nickel sulfide particle at the top. Notably, the CNT shows a well-defined tubular structure and graphitic crystallization, even though the lattice spacing is a little larger (0.35–036 nm).

**Fig. 6:**
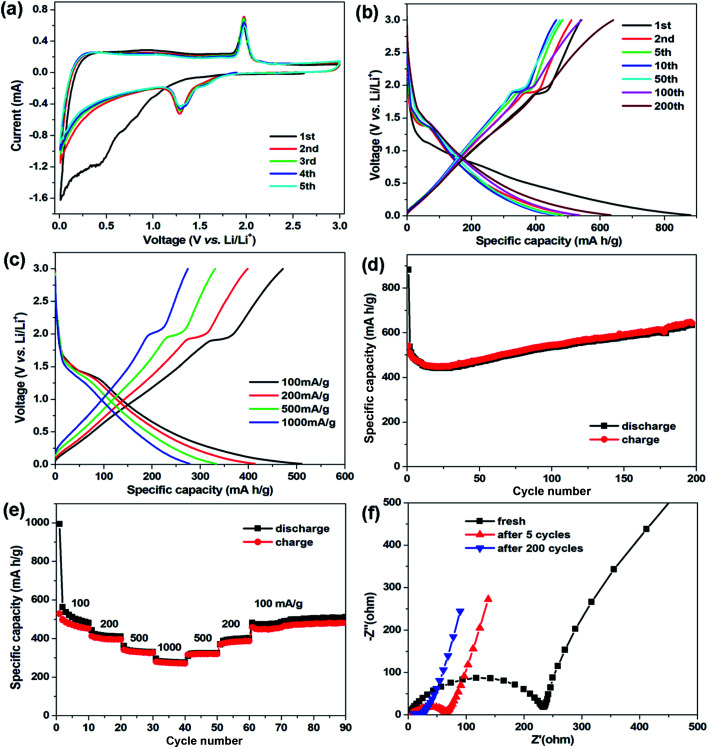
Electrochemical properties of CNT@NS@CNFs. (a) CV curves at a scan rate of 0.2 mV s^−1^; (b) galvanostatic discharge–charge profiles at 100 mA g^−1^; (c) galvanostatic discharge–charge profiles at different current densities; (d) cycle performance at 100 mA g^−1^; (e) rate performance at currents ranging from 100 to 1000 mA g^−1^; and (f) Nyquist plots of the CNT@NS@CNF electrode at different cycle stages.

The electrochemical properties of the CNT@NS@CNF hybrid were studied in coin-type cells using lithium foil as the counter and reference electrodes. Fig. 6a displays the cyclic voltammetry (CV) curves of the CNT@NS@CNF electrode at 0.2 mV s^−1^ in 0.01–3.0 V. In the initial cathodic scan, the broad peak below 1.0 V along with the weak peak at 0.43 V can be attributed to the formation of the solid-electrolyte interphase (SEI) and the conversion reaction.^37^ In the following cycles, a pair of redox peaks can be clearly observed at about 1.28/1.97 V, corresponding to the lithiation/delithiation of Ni_3_S_2_ (Ni_3_S_2_ + 4Li^+^ + 4e^−^ → 3Ni + 2Li_2_S), whose theoretical capacity is 445 mA h g^−1^.^18^ In addition, the CV curves are well overlapped from the second to the fifth cycle, indicating the highly reversible reactions and the good cycling stability of the CNT@NS@CNF electrode during the discharge–charge process.

Fig. 6b shows the galvanostatic discharge–charge profiles of the CNT@NS@CNF electrode in different cycles at 100 mA g^−1^. The voltage plateau at ∼1.3 V in the charging process can still be clearly observed, which is in agreement with the CV results. The initial discharge/charge capacities are 881.5/539.5 mA h g^−1^ with an initial coulombic efficiency of 61.2%. The large capacity loss is mainly due to the formation of the SEI layer and decomposition of the electrolyte in the first cycle.^38^ To further investigate the capacity–rate relationship of CNT@NS@CNFs, the discharge/charge voltage profiles at various current densities are given in Fig. 6c. Two plateaus can still be clearly observed when the current increases from 100 mA g^−1^ to 1000 mA g^−1^, indicating the high rate capability and the efficient lithium reaction kinetics in the hybrid electrode.

Fig. 6d shows the cycling performance of the CNT@NS@CNF electrode at 100 mA g^−1^. In the initial few cycles, the specific capacities slowly decrease and then steadily increase in the following cycles, which can be attributed to the full activation of the electrode materials,^17^ as the nickel sulfide nanoparticles are fully encapsulated within the graphitic carbon shells. The CNT@NS@CNF hybrid electrode delivers a high reversible capacity of 633 mA h g^−1^ after 200 cycles with the coulombic efficiency approaching 100%. The increased capacities could be attributed to the destruction in the graphite lattice during the insertion and extraction of lithium ions and the generation of more defects and active sites for lithium-ion storage.^18^ Note that Ni_3_S_2_ shows a theoretical capacity of 445 mA h g^−1^, the capacity contribution of NS with a content of ∼53% in the CNT@NS@CNFs is ∼236 mA h g^−1^ (633 mA h g^−1^ × 0.53), and thus the capacity of CNF–CNT is as high as ∼845 mA h g^−1^. Fig. 6e shows the rate performance of the CNT@NS@CNF electrode, which delivers reversible capacities of 516, 413, 334 and 277 mA h g^−1^ each after 10 cycles at 100, 200, 500 and 1000 mA g^−1^, respectively. When recycled at 100 mA g^−1^, a high reversible capacity of 510 mA h g^−1^ is still retained after another 30 cycles. Moreover, the effects of the NiAc_2_ amount and temperature on the structural characteristics as well as the lithium storage performance of CNT@NS@CNFs were also investigated and discussed in the ESI (Fig. S4 and S5).†

To investigate the charge transfer and Li^+^ diffusion kinetics of the CNT@NS@CNF electrode, electrochemical impedance spectroscopy (EIS) measurement was carried out, and Fig. 6f shows the Nyquist plots of the fresh CNT@NS@CNF electrode and its counterparts after different cycles. The semicircle in the high-frequency region is related to the charge-transfer process and the straight line in the low-frequency region is normally related to a semi-infinite Warburg diffusion process.^39^ The fresh CNT@NS@CNF electrode exhibits a charge transfer resistance of ∼233 Ω, which greatly decreases when increasing the cycle number from the 5^th^ to the 200^th^ cycle. The steeper inclined line at low frequency indicates the faster lithium ion diffusion, which also demonstrates the superior electrochemical performance of the CNT@NS@CNF hybrid.^13^

**Fig. 7:**
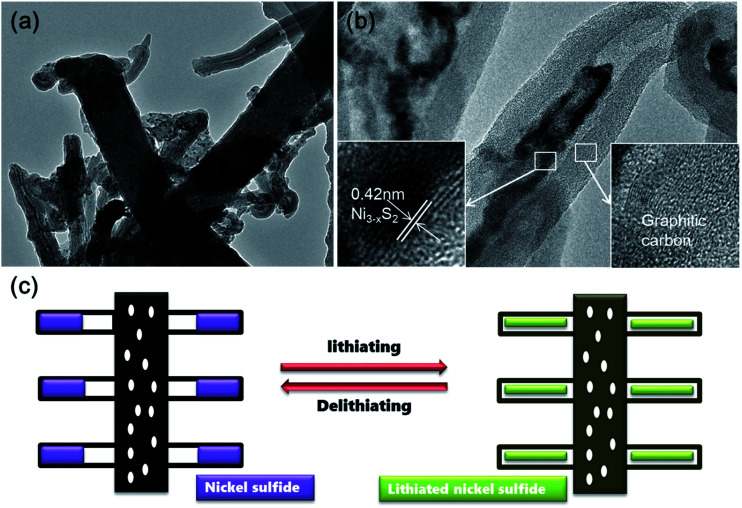
(a) TEM image of the CNT@NS@CNFs after cycling in the charged state and (b) TEM image of a CNT capped with NS with the insets showing the HRTEM images taken from the selected zones correspondingly. (c) Schematic illustration of the CNT@NS@CNF upon lithium insertion/extraction.

In order to investigate the structural evolution upon cycling, *ex situ* TEM analysis was performed on the CNT@NS@CNF electrodes after 200 cycles at 100 mA g^−1^. As shown in Fig. 7, the CNT and CNF morphologies are well maintained after cycling. Notably and interestingly, the nickel sulfide nanoparticles are still fully encapsulated within the CNTs, indicating that the lithium insertion/extraction within nickel sulfides and the accompanying volume expansion are well confined within the CNTs, which synergistically contribute to the superior cycling stability. Therefore, the superior cycle stability and rate capability of the hierarchical CNT@NS@CNFs can be attributed to the novel structural characteristics on several aspects. First, the encapsulation of nickel sulfide nanoparticles within the graphitic CNT/CNFs not only efficiently buffers the volume changes upon lithiation/delithiation, but also facilitates charge transfer and electrolyte diffusion owing to the highly conductive networks with open frame structures.^17,18,38^ Secondly, N- and S-doping into the carbon matrices could produce localized highly reactive regions and improve the kinetics of the lithium-ion diffusion-transfer.^18,40^ Moreover, Table S1† summarized the structures, synthesis methods and electrochemical properties of various nickel sulfides in the previous literature, which further reveals the superiority of the current hierarchical CNT@NS@CNFs when examined as anode materials for lithium-ion batteries.

## 4. Conclusions

In summary, we have demonstrated the facile fabrication of hierarchical CNT@NS@CNF architectures *via* a combined strategy of electrospinning and the CVD method. Under an Ar/thiophene flow at high temperature, CNTs were readily grown out of the CNFs with the catalytic effect of the metallic Ni species, which arose from the carbonization of NiAc_2_/PAN. At the same time, the Ni particles were sulfurized into nickel sulfides which were fully encapsulated into the CNTs. Owing to the novel hybrid architecture, the dendritic CNT@NS@CNFs showed superior cycle stability and rate capability, delivering a high reversible capacity of 630 mA h g^−1^ at 100 mA g^−1^ after 200 cycles and 277 mA h g^−1^ at a high rate of 1000 mA g^−1^, when examined as LIB anode materials. More importantly, this novel CVD-sulfurization method can be generally applied to fabricate other carbon-supported metal (*e.g.*, Co, Fe) sulfides (Fig. S6†) for application in energy storage and conversion fields.

## Conflicts of interest

There are no conflicts to declare.

## Supplementary Material

NA-001-C8NA00234G-s001
