# Supplementary material for: Chemical vapor deposition growth of carbon nanotube confined nickel sulfides from porous electrospun carbon nanofibers and their superior lithium storage properties
Source: Nanoscale Adv. 2018 Oct 12;1(2):656–63. doi: 10.1039/c8na00234g (PMC9473167; doi:10.1039/c8na00234g)
Supplement: NA-001-C8NA00234G-s001 [file NA-001-C8NA00234G-s001.pdf]

Electronic Supplementary Material (ESI) for Nanoscale Advances.
This journal is © The Royal Society of Chemistry 2018

Supporting information

Chemical Vapor Deposition Growth of Carbon Nanotube Confined Nickel Sulfides from Porous Electrospun Carbon Nanofibers and Their Superior Lithium Storage Properties

An Wang,[a] Sanmu Xie,[a] Rong Zhang,[a] Yiyi She,[b] Chuan Chen,[c] Micheal K H Leung,[b] Chunming Niu,[a] Hongkang Wang[a,*]

[a] *State Key Lab of Electrical Insulation and Power Equipment, Center of Nanomaterials for Renewable Energy (CNRE), School of Electrical Engineering, Xi'an Jiaotong University, Xi'an 710049, People's Republic of China. E-mail: hongkang.wang@mail.xjtu.edu.cn*

[b] *Ability R&D Energy Research Centre (AERC), School of Energy and Environment, City University of Hong Kong, Hong Kong SAR, People's Republic of China.*

[c] *Global Energy Interconnection Research Institute co., Ltd., Future Science Park, Changping district, Beijing 102211, People's Republic of China.*

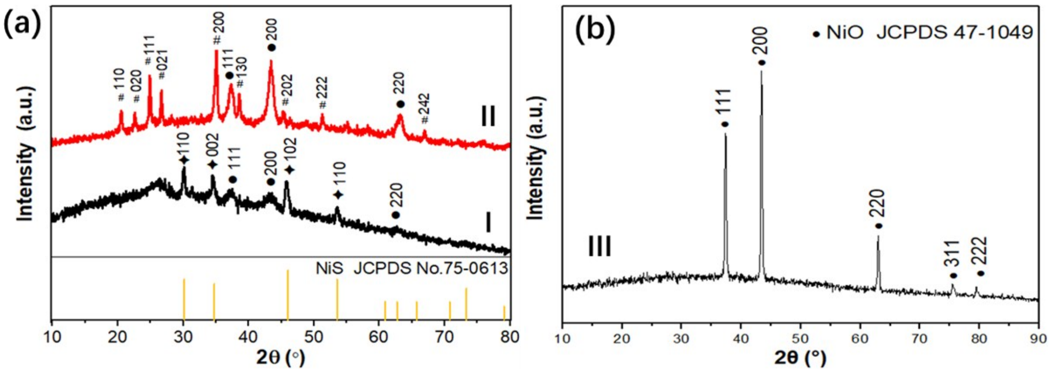

Figure S1. XRD patterns of the intermediates collected at different temperatures during TGA measurement. (a-I) 480 °C; (a-II) 630 °C; (b) 800 °C. Notes: NiS (✦), NiO (●), $NiSO_4$ (#) .

XRD analysis was performed on the products obtained by stopping the TGA analysis at different temperatures (Figure S1). The product collected at 480 ℃ is a mixture of NiS and NiO. In the product collected at 630 ℃, only NiO and $NiSO_4$ were

detected. The product collected at 800 ℃ is NiO. Based on the above results, the oxidation of nickel sulfides (Ni_3S_2) in the CNT@NS@CNF hybrid under the TGA analysis can be proposed as following:

$$2Ni_3S_2 + O_2 \rightarrow 4NiS + 2NiO \quad (1)$$

$$NiS + 2O_2 \rightarrow NiSO_4 \quad (2)$$

$$2NiSO_4 \rightarrow 2NiO + 2SO_2 + O_2 \quad (3)$$

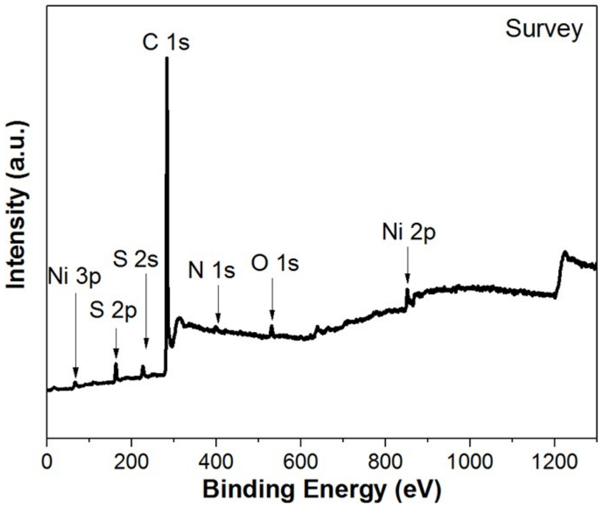

Figure S2. Survey XPS spectrum of CNT@NS@CNFs.

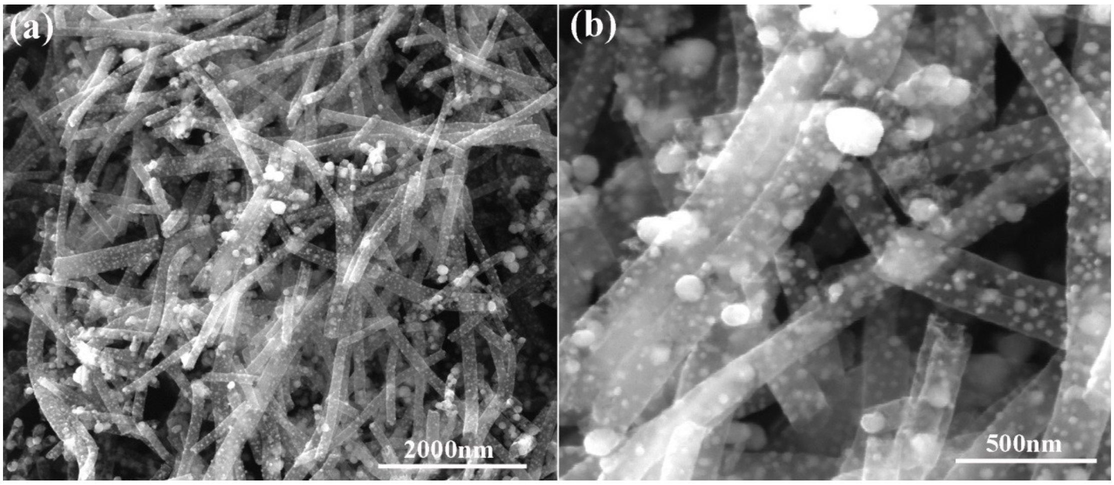

Figure S3. SEM images of the Ni@CNFs obtained by annealing $NiAc_2$/PAN precursor at 800 ^oC with the absence of thiophene.

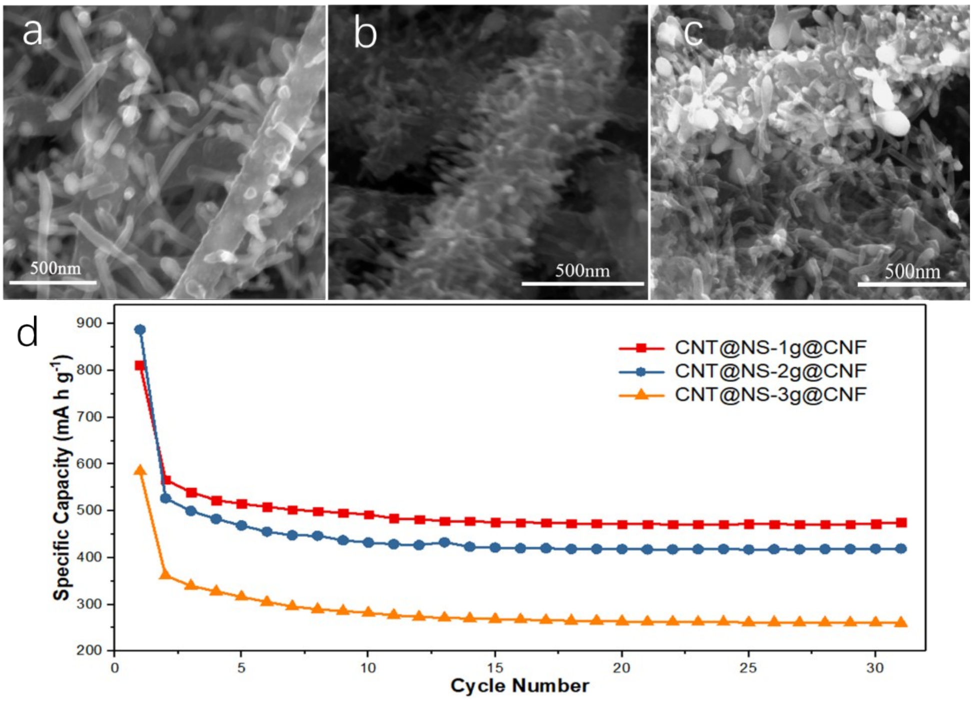

Figure S4. (a-c) SEM images and (d) cycling performances of (a) CNT@NS-1g@CNFs, (b) CNT@NS-2g@CNFs and (c) CNT@NS-3g@CNFs, in which the 1g, 2g and 3g represent the introduction amount of $NiAc_2$ precursor in the electrospun solution.

To explore the intrinsic factors influencing the morphological and electrochemical characteristics of the CNT@NS@CNFs, a series of controlled experiments were conducted. With an increase of the introduction amount of $NiAc_2$ in the electrospun solution, the nanotubes increase in number and size owing to the increased Ni nanoparticles (Figure S3). However, with increasing the amount of $NiAc_2$ to 2g and 3g in precursor, the as-obtained electrodes even showed worse lithium storage performance, delivering discharge capacities of 411 and 260 mA h/g after 45 cycles at a current density of 100 mA/g, respectively (Figure S3d), which can be attributed to the lower theoretical specific capacity of nickel sulfides. This may also support that the hierarchical CNT-CNF architectures contributed a lot capacity owing to the novel structure characteristics.

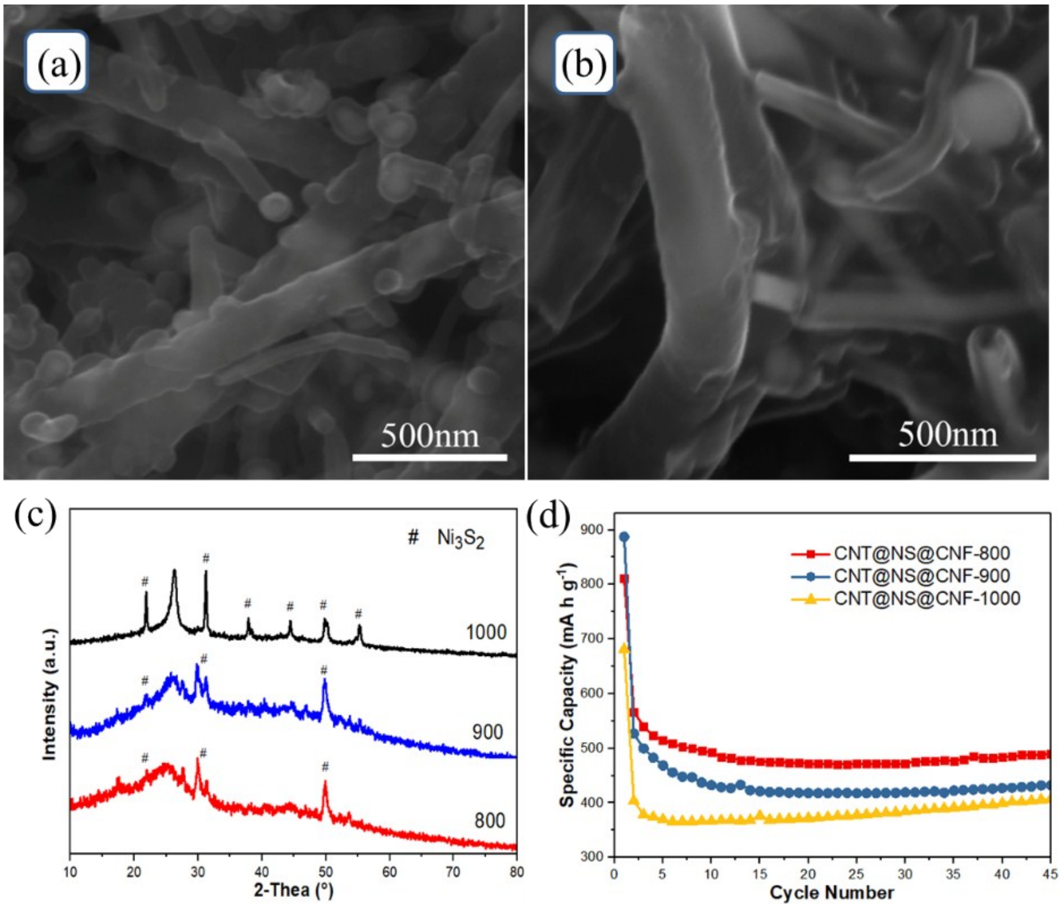

Figure S5. (a, b) SEM images of (a) CNT@NS@CNF-900 and (b) CNT@NS@CNF-1000. (c) XRD patterns and (d) cycling performances of the CNT@NS@CNFs prepared at different temperatures (the number represents the reaction temperatures).

Temperature-depended experiments were also performed, and the results were shown in Figure S4. When annealing the precursor $NiAc_2$/PAN nanofibers at 900℃ and 1000 ℃, the nickel sulfide nanoparticles encapsulated inside the tubes changed from ellipsoids to spherical shapes and their size increased with the temperature. As shown in Figure S4c, XRD pattern of CNT@NS@CNFs-800, CNT@NS@CNFs-900 and CNT@NS@CNFs-1000 were compared, revealing the increased crystallinity with increasing annealing temperature. When examined as anode materials for lithium ion batteries, the CNT@NS@CNFs-900, CNT@NS@CNFs-1000 exhibited discharge capacities of 432 mA h/g and 399 mA h/g after 45 cycles at a current density of 100 mA/g. In addition, the reaction time is also an important factor. The CNT@NS@CNFs-1h (obtained by annealing at 800℃ for 1h) only showed a discharge capacities of 244 mA h/g after 10 cycles at 100 mA/g. All these products

have a problem of the aggregation of Ni_3S_2. When the reaction time is double, the Ni_3S_2 nanoparticles were easier to grow bigger. Higher reaction temperature produced larger Ni_3S_2 particles inside the carbon fibers owing to the fast nuclear and growth, which caused the inferior mechanical stability and the poorer lithium storage properties.

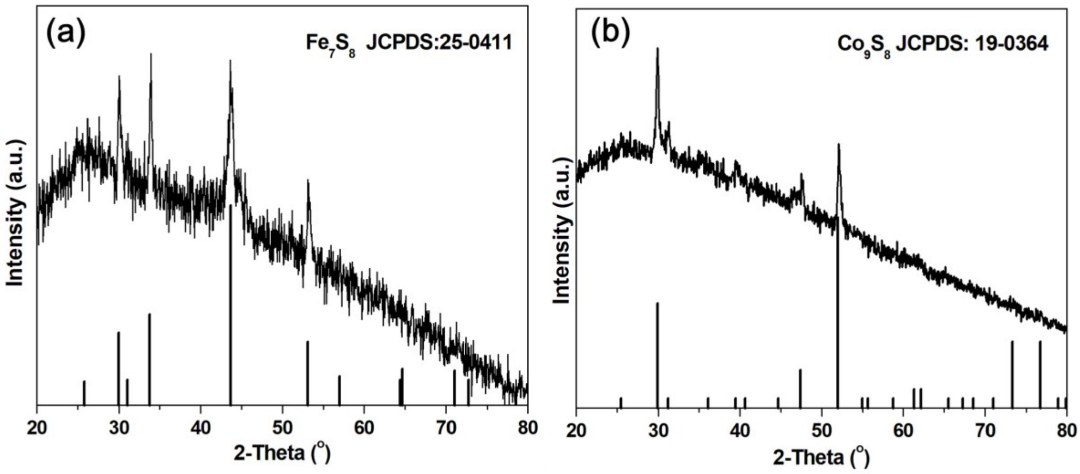

Figure S6. XRD patterns of iron sulfide and cobalt sulfide obtained using thiophene as sulfur source via CVD method, in which cobalt acetate or iron nitrate were used as Fe or Co sources for the electrospinning.

Table1. Structures, electrochemical properties and synthesis methods of nickel sulfides as anode materials for lithium ion batteries

Structure	Electrochemical properties	Synthesis method	Ref
NiO/Ni_3S_2-CNF composites	519.2mAh/g after 200 cycles at 0.5 A /g	$Ni(NO_3)_2·6H_2O$, urea and SDS into distilled water as precursor; hydrothermal reaction at 180°C for 12 h; annealing at 500°C in N_2 atmosphere for 2 h	1
kiwano-like hollow structure NiS_2 electrode	681 mA h/g after 100 cycles at 50 mA /g	$NiCl_2·6H_2O$, $N_2H_4·H_2O$ and NaOH dissolved into ethylene glycol as precursor; hydrothermal reaction at 120°C for 2 h, the product is mixed with sulfur and maintained at 170°Cfor 10h	2
NS@CNT electrode	644 mA h/ g after 100 cycles at 0.3A/g.	nickel chloride hexahydrate and thiourea as electrodeposition solution, a piece of CNT placed	3

		into the electrolyte for electrodeposition	
CMK-3-Ni_3S_2 composites	~520 mAh/g after 100 cycles at 0.1A/g	Sucrose, SBA-15,$Ni(NO_3)_2 \cdot 6H_2O$ and H_2SO_4 dissolved in deionized water as precursor solution; annealing at 700℃for 3h in N_2 atmosphere and wash by NaOH solution	[4]
CNTs@C@NiS electrodes	649mAh/g after 100 cycles at 0.1A/g	CNTs and glucose heated at 180 °C for 6 h in Teflon-lined autoclave, the product mixed with $NiCl_2.6H_2O$, thiourea and glucose for hydrothermal reaction at 180℃ for 12 h	[5]
Ni_3S_2 nanosheet array	660.9mAh/g after 60 cycles at 0.1A/g	$Na_2S_2O_3 \cdot 5H_2O$ and Na_2SO_4 solution ,Ni foam for hydrothermal reaction at 130℃ for 2 h	[6]
CNT@NS@CNFs	630mAh/g after 200 cycles at 100mA/g	PAN/$NiAc_2$ nanofibers annealing at 800℃ for 30min in thiophene atmosphere	***This work***

References.

1. J. Jiang, C. Ma, Y. Yang, J. Ding, H. Ji, S. Shi and G. Yang, *Appl. Surf. Sci.*, 2018, 441, 232-238.
2. Y. Zhang, F. Lu, L. Pan, Y. Xu, Y. Yang, Y. Bando, D. Golberg, J. Yao and X. Wang, *J. Mater. Chem. A*, 2018, 6, 11978-11984.
3. P. Fan, H. Liu, L. Liao, J. Fu, Z. Wang, G. Lv, L. Mei, H. Hao, J. Xing and J. Dong, *RSC Advances*, 2017, 7, 49739-49744.
4. S. Zhang, R. Lin, W. Yue, F. Niu, J. Ma and X. Yang, *Chem. Eng. J.*, 2017, 314, 19-26.
5. R. Jin, Y. Jiang, G. Li and Y. Meng, *Electrochimica Acta*, 2017, 257, 20-30.
6. Y. Wang, Y. Niu and C. M. Li, *ChemistrySelect*, 2017, 2, 4445-4451.
